# The mitochondrial enzyme FAHD1 regulates complex II activity in breast cancer cells and is indispensable for basal BT‐20 cells *in vitro*

**DOI:** 10.1002/1873-3468.14462

**Published:** 2022-08-12

**Authors:** Max Holzknecht, Lena Guerrero‐Navarro, Michele Petit, Eva Albertini, Elisabeth Damisch, Anna Simonini, Fernando Schmitt, Walther Parson, Heidelinde Fiegl, Alexander Weiss, Pidder Jansen‐Duerr

**Affiliations:** ^1^ Institute for Biomedical Aging Research Leopold‐Franzens University of Innsbruck Austria; ^2^ Medical Faculty, CINTESIS@RISE (Health Research Network), Alameda Prof. Hernâni Monteiro University of Porto Portugal; ^3^ Institute of Legal Medicine Medical University of Innsbruck Austria; ^4^ Forensic Science Program The Pennsylvania State University University Park PA USA; ^5^ Department of Obstetrics and Gynaecology Medical University of Innsbruck Austria

**Keywords:** FAHD1, glutaminase, MCF‐7, oxaloacetate, succinate dehydrogenase, TNBC

## Abstract

The mitochondrial enzyme fumarylacetoacetate hydrolase domain‐containing protein 1 (FAHD1) was identified to be upregulated in breast cancer tissues. Here, we show that FAHD1 is indispensable for the survival of BT‐20 cells, representing the basal breast cancer cell type. A lentiviral knock‐down of FAHD1 in the breast cancer cell lines MCF‐7 and BT‐20 results in lower succinate dehydrogenase (complex II) activity. In luminal MCF‐7 cells, this leads to reduced proliferation when cultured in medium containing only glutamine as the carbon source. Of note, both cell lines show attenuated protein levels of the enzyme glutaminase (GLS) which activates programmed cell death in BT‐20. These findings demonstrate that FAHD1 is crucial for the functionality of complex II in breast cancer cells and acts on glutaminolysis in the mitochondria.

## Graphical abstract

Depletion of the mitochondrial enzyme FAHD1 in breast cancer cells leads to metabolic stress and cell death in triple‐negative BT‑20 cells. We find indications for reduced complex II (succinate dehydrogenase) activity and attenuated glutaminase levels upon knockdown of FAHD1. These effects are relevant in glutamine‐affine cells, such as triple‐negative breast cancer cells, whereas luminal MCF‐7 cells tolerate the withdrawal of FAHD1 to a certain extent.

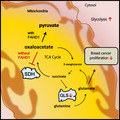

## Abbreviations

**2‐DG**, 2‐deoxy‐glucose

**ACT**, actin

**ADP**, adenosine‐di‐phosphate

**Ama**, antimycin A

**BPTES**, Bis‐2‐(5‐phenylacetamido‐1,3,4‐thiadiazol‐2‐yl)ethyl sulfide

**CI‐CV**, complex I to V of the respiratory chain

**cPDL**, cumulative population doublings

**DMEM**, Dulbecco's modified eagle's medium

**ECAR**, extra cellular acidification rate

**FAHD1**, fumaryl‐acetoacetate‐hydrolase‐Domain containing protein 1

**FCS**, fetal calf serum

**GAC**, glutaminase C

**GAPDH**, glyceraldehyde‐3‐phosphate dehydrogenase

**GLS**, glutaminase

**GLUL**, glutamine synthase (Gene)

**GS**, glutamine synthase

**HUVEC**, human umbilical vein endothelial cells

**IHC**, immunohistochemistry

**kDa**, kilo‐Dalton

**KGA**, kidney‐like glutaminase isoform

**m**, molar

**MiR05**, mitochondrial respiration medium 05

**mm**, millimolar

**MOI**, multiplicity of infection

**mPEPCK**, mitochondrial phosphoenol‐pyruvate‐carboxy‐kinase

**NADH**, nicotinamidadenindinukleotid

**NIH**, National Institutes of Health, US

**nm**, nanomolar

**ns**, non‐significant

**O2K**, oxygraph‐2k

**OAA**, oxaloacetate

**OCR**, oxygen consumption rate

**ODx**, oxaloacetate decarboxylation

**PAGE**, polyacrylamid‐gelelectrophoresis

**PBS**, phosphate‐buffered saline

**PC**, pyruvate‐carboxylase

**PEP**, phosphoenol‐pyruvate

**SCR**, scrambled

**SDH**, succinate‐dehydrogenase

**SDS**, safety data sheet or sodium dodecyl sulfate

**SEM**, standard error of the mean

**SOP**, standard operating procedures

**TNBC**, triple‐receptor negative breast cancer

**WT**, wild‐type

μ **m**, micromolar

Traditionally, glucose was considered the most important carbon source for rapidly dividing cancer cells [1]. Different metabolites and intermediates, mostly provided by the breakdown of glucose, are consumed by tumour cells and integrated in anaplerotic reactions for cellular proliferation under hypoxic conditions. Resulting pyruvate is mainly converted to lactate, as first hypothesized by Otto Warburg [2]. Besides glucose, the import [3] and usage of the amino acid glutamine for metabolic function in cancer cells gains more attention [4]. Glutamine is also a precursor for the biosynthesis of macromolecules such as nucleotides, proteins [5] and lipids [6]. It can fuel the TCA cycle *via* glutaminase activity or provide glutamate for extracellular signalling [7]. In addition, glutamate can be converted to alpha‐ketoglutaric acid which can impact histone demethylation patterns and regulate cellular pluripotency [8, 9] with potential implications in cancer [10, 11]. Especially triple‐negative breast cancer cells (TNBC) are prone to rely on glutamine as the main carbon source [12]. Overexpression of glutaminase (GLS) makes these cell types sensitive to treatments that target this metabolic route [13]. Inhibitors of GLS such as Bis‐2‐(5‐phenylacetamido‐1,3,4‐thiadiazol‐2‐yl)ethyl sulfide (BPTES) [14] and its derivatives (e.g. CB‐839) have been promising candidates in clinical trials in the recent past [15]. Tumours that have a more luminal and hormone‐receptor positive phenotype could overcome this vulnerability by expressing genes that provide glutamine as product (Glutamine synthase, GS; *GLUL*) [16] and therefore balance the metabolic flux between glycolysis and glutaminolysis. The detailed regulations of these pathways are still not fully understood which makes the development of new therapeutic options challenging, especially for TNBC [17].

The activity of the mitochondrial enzyme FAHD1 contributes to the regulation of oxaloacetate (OAA) levels through decarboxylation (oxaloacetate decarboxylation, ODx) [18] yielding pyruvate as product. It is hypothesized that the activity of FAHD1 as ODx is important to balance TCA cycle intermediate levels and allow unhindered flux through the respiratory chain [19, 20]. In human umbilical vein endothelial cells (HUVEC), FAHD1 KD caused impaired mitochondrial respiration, reduced ATP production and lower mitochondrial membrane potential [21]. Interestingly, FAHD1‐KO in C57/BL6 mice resulted in accumulation of OAA in both liver and kidney [18]. These findings support the idea that FAHD1 is regulating levels of oxaloacetate in mitochondria. Such a metabolic activity is unique for eukaryotic enzymes and was described only a few years ago [18]. This study aims to further characterize the metabolic function of FAHD1 in breast cancer and to contribute new insights into the regulatory network that depends on its ODx activity in the TCA cycle. Here, we present a model for highly specialized cell types that rely on glutaminolysis to maintain proliferative capacity such as found in TNBC.

## Material and methods

### Cell culture conditions

The luminal breast cancer cell line MCF‐7 was cultured in DMEM media (Pan‐Biotech, Aidenbach, Germany) supplemented with 15 mm glucose (2.5 m Stock, Sigma, St. Louis, MO, USA), 4 mm glutamine (200 mm Stock, Sigma), 100 μm pyruvate (100 mm Stock, Sigma), 1× Penicillin/Streptomycin (Sigma) and 10% FCS (Gibco, Thermo Fisher Scientific, Waltham, MA, USA) for culture expansion and regular cultivation. For limiting media conditions, either glucose or glutamine was omitted and normal FCS was replaced by 10% dialysed FCS (Thermo) to avoid glucose or glutamine traces in the non‐dialysed serum. The basal breast cancer cell line BT‐20 was cultured in EMEM (Lonza, Basel, Switzerland) supplemented with 4 mm glutamine, 1× non‐essential amino acids (Pan‐Biotech), 1× Penicillin/Streptomycin and 5% FCS. The culture medium was changed every other day. At 90% confluence, cells were detached with trypsin (Sigma), split and reseeded on either 10 cm dishes (Sigma), T‐75 (Sarstedt, Nümbrecht, Germany) or T‐175 flasks (Sarstedt) during expansion periods. Breast cancer cell lines used for FAHD1 expression analysis were grown in DMEM (Pan‐Biotech) supplemented with glucose (15 mm), glutamine (4 mm), pyruvate (100 μm), 10% FCS and 1× Penicillin/Streptomycin at 37 °C in the presence of 5% CO_2_. In order to authenticate the cell lines, MCF‐7, T47D, BT‐20 and MDAMB‐231, the amplification of 15 STR loci and the sex‐specific locus amelogenin was carried out at the Institute of Legal Medicine, Medical University of Innsbruck [22].

### Lentiviral infection and shRNA‐mediated knock‐down

Viral particles were produced using the psPAX packaging plasmid (Addgene, Cambridge, MA, USA, #12260), the pMD2.G envelope plasmid (Addgene, #12259) and the lentiviral plasmid of interest. The target sequence for the most efficient FAHD1 KD was AGAUGAACCCUUCAAGAAA; the SCR control plasmid was purchased from Addgene. Both plasmids carry a puromycin resistance to select infected cells at 0.5 μg·mL^−1^ puromycin throughout the experimental procedure. All cells were infected with 10 multiplicity of infection (MOI) by default. Cell lines were incubated with the respective virus for 24 h in the presence of 8 μg·mL^−1^ polybrene (Sigma) before the medium was changed to regular cultivation conditions under selection with puromycin. Knock‐down efficiency was tested *via* qPCR and western blotting.

### Growth curves

After the expansion of the respective cell line, 1.5 × 10^5^–2.5 × 10^5^ cells were seeded in 2 mL final volume on two six‐well plates with three control wells (SCR) and three wells containing FAHD1 KD cells on each plate. Once the control cells reached 90% confluency, cells were detached and counted with the CASY cell counter system (Schärfe System). Three data points were obtained for SCR and FAHD1 KD cells for the respective days. Cumulative population doubling (cPDL) was calculated [23]. Arithmetic mean cPDL values of each experimental day were summed. The error bars show the *standard error of the mean* (SEM). Cells were then reseeded at equal density and grown until they reached confluency again. The medium was changed every other day for the duration of the experiment.

### Western blotting

Protein extraction was obtained by lysing 1.0 × 10^6^ cells in 200 μL RIPA‐buffer (containing protease inhibitors) with 10–15 ultra‐sound pulses. The protein concentration was measured *via* a BCA‐Assay (Pierce BCA Protein Assay Kit, Thermo) using BSA‐based standards. Each sample was mixed with 4× SDS sample buffer and heated to 95 °C for 5 min. For gel electrophoresis, 30 μg of total protein was loaded on a 12.5% acrylamide mini‐gel to separate the proteins according to their molecular weight by SDS/PAGE (Mini‐PROTEAN^®^ Tetra Cell, Bio‐Rad, Hercules, CA, USA). The pre‐stained PAGE‐Ruler (PageRuler™ Prestained Protein Ladder, Thermo‐Scientific) was used as a marker. The proteins were blotted on a PVDF‐Membrane (Immun‐Blot^®^, Bio‐Rad) in 1× transfer buffer (10% methanol) for 1 h at 300 mA_const_ and around 100 V. The membrane was blocked with 5% skim milk (in PBS + 0.1% Tween) for 2 h, cut into slices at the respective height and incubated with the primary antibodies (in 5% milk) overnight. The secondary antibody was applied for 45–120 min, dependent on the antigen. After several washing steps in PBS‐T and PBS, the membrane was reassembled and incubated for 2 min in luminol developer solution (Immonilon^®^ Western, HRP Substrate, Millipore, Burlington, MA, USA) before imaging with the ChemiDoc™ Imaging System (Bio‐Rad).

### Densitometric evaluation of protein expression

Pictures in .*tif* format were analysed using the imagej software (*ImageJ* open source, NIH). For each sample on the blot, equal areas were chosen to measure the *mean grey value* of the given band. Values were inverted (= 255 – value) and the background was subtracted for each band. Relative intensities were calculated using a structural protein as loading control (e.g. Actin or Tubulin). Columns show the arithmetic mean with the SEM.

### Flow cytometry

Induction of programmed cell death (apoptosis) was tested using the FACS Canto (BD‐Biosciences, Franklin Lakes, NJ, USA). 2.0 × 10^5^ BT‐20 cells were harvested and analysed at day 7 and day 8 post‐infection. Staining with Annexin V/PI (BD‐Biosciences) was performed for 30 min at 37 °C. Cells were washed with PBS and analysed by flow‐cytometry. Unstained cells were used as controls to check for auto‐fluorescence and channel spillover. Dead cells and cellular debris were excluded from the analysis (SSC/FSC; see Fig. S4A). Annexin V‐positive cells were assumed to be early apoptotic cells and percentages of this population were calculated using the flowjo software (BD‐Biosciences). For each day, *n* = 3 biological replicates were analysed.

### High‐resolution respirometry

Oxygen consumption rates (OCR) of MCF‐7 and BT‐20 cells were measured with two Oxygraph‐2k respirometers (O2k, Oroboros GmBH, Innsbruck, Austria) in parallel (four chambers with 2× SCR and 2× FAHD1 KD). Cells were detached with trypsin, washed in PBS and counted with the CASY cell counter. For each measurement, 5.0 × 10^5^–7.5 × 10^5^ cells were loaded to the chambers containing 2 mL medium (Mitochondrial respiration medium 05, MiR05, see https://wiki.oroboros.at/index.php/MiR05) warmed to 37 °C. The employed protocol (SUIT‐010) focuses on complex II (succinate dehydrogenase) activity and was slightly adapted from *Bioblast*.*at*. Basal oxygen consumption in the absence of any substrates was obtained after adding the cells to the chamber. Subsequently, complex I (NADH–ubiquinone‐oxidoreductase) was inhibited by rotenone (500 nm) and complex II was stimulated by supplying succinate (25 mm) and ADP (2.5 mm final). The cellular membrane was then permeabilized by a stepwise titration of digitonin (1 μg·μL^−1^, see https://wiki.oroboros.at/index.php/Digitonin) with an average volume of 4–5 μL per chamber. The following inhibition of complex II by malonic acid (10 mm) led to a decrease in mitochondrial oxygen consumption and was used to calculate specific complex II activity. Final inhibition of complex III (cytochrome‐c‐reductase) with Antimycin A (2.5 μm) decreased the signal to background levels. In order to compare mitochondrial function of SCR and FAHD1 KD cells, each data point was normalized for basal OCR rates at the beginning of the measurement. These normalized values are called *flux control ratio* (FCR, see https://wiki.oroboros.at/index.php/Flux_control_ratio) and represent OCR levels at different time points relative to initial basal OCR levels in each chamber. For MCF‐7 in *full medium n* = 6 measurements and in limiting conditions *glutamine only* and *glucose only n* = 4 independent measurements are depicted respectively.

### Glycolytic flux analysis

Based on extracellular acidification rates, glycolytic activity of MCF‐7 and BT‐20 cells was measured with the *Seahorse XF HS Mini* Metabolic Flux Analyzer (Agilent, Santa Clara, CA, USA). For each experiment, two 8‐well cell plates containing 3× SCR and 3× FAHD1 KD with two blank wells were used. In short, the cells were seeded the day before the experiment at 2.0 × 10^4^ cells per well in DMEM pH 7.4 (Agilent) supplemented with glutamine, pyruvate and glucose and kept at 37 °C in an incubator with 5% CO_2_. One hour before the measurement, the medium was changed to the respective assay conditions (according to Agilent's *Glycolytic Rate* or *Glycolytic Stress Test*) and moved to a CO_2_‐free incubator for 45–60 min. Meanwhile, the sensor cartridges were loaded with the respective chemicals according to the assay (Glucose, oligomycin, 2‐deoxy‐glucose; rotenone/antimycin A, 2‐deoxy‐glucose). The cell plates were loaded into the *Seahorse HS Mini* and extracellular acidification rates (ECAR) were detected as mpH·min^−1^. To normalize each measurement to the amount of biological material, either seeded cell numbers were considered or total protein levels of each well were measured *via* a Bradford Assay (Table 1).

**Table 1:** List of chemicals and antibodies all chemicals were used according to standard operating procedures (SOP) and the safety data sheet provided by the company.

	Company	Cat. number
Cell culture		
DMEM, 500 mL	Pan‐Biotech	P04‐01548
DPBS, 500 mL	Pan‐Biotech	P04‐36500
EMEM, 500 mL	Lonza	12‐125F
Trypsin, 100 mL	Sigma	T3924
FCS, 500 mL	Gibco	10270‐106
Dialysed FCS, 500 mL	Thermo	26400044
D‐Glucose, 100 mL, 2.5 m	Sigma	G8769
L‐glutamine, 100 mL, 200 mm	Sigma	G7513
Penicillin/Streptomycin, 100 mL	Sigma	P4333
MEM non‐essential amino acids, 100 mL	Pan‐Biotech	P08‐32100
Dimethylsulfoxide (DMSO)	Roth	4720.4
Sodium pyruvate, 100 mL, 100 mm	Sigma	S8636
Polybrene, 5 g	Sigma	H9268
Puromycin, 10 mg	Sigma	P8833
10 cm dish	Sarstedt	83.3902
6‐well plate	Sarstedt	83.3920
T175 flask	Sarstedt	83.3912
T75 flask	Greiner	658170
CASY^®^ cell counter and analyser	Schärfe System	Model DT
High‐resolution respirometry, O2K		
Malonic acid, 5 g	Sigma	M1296
di‐Sodium succinate, 100 g	Sigma	8.18601
ADP sodium salt, 5 × 1 g	Sigma	A2754
Digitonin	Sigma	D141
Rotenone	Sigma	R8875
Antimycin A	Sigma	A8674
Seahorse XF HS Mini		
XF Calibrant Solution, 100 mL	Agilent	103059‐000
XF DMEM Medium pH 7.4, 500 mL	Agilent	103575‐100
Seahorse XF HS Mini FluxPak	Agilent	103723‐100
Glycolytic Stress Test Kit	Agilent	103017‐100
Glycolytic Rate Assay Kit	Agilent	103346‐100
Wet lab		
Skim milk powder, 500 g	Sigma	70166
Tween^®^ 20, 100 mL	Sigma	P1379
Magnesiumchloride‐Hexahydrate, 1000 g	Merck	1.05833.1000
Potassium‐dihydrogen‐phosphate, 1000 g	Roth	3904.1
HEPES, 250 g	Sigma	H3375
Taurine, 100 g	Sigma	T0625
Lactobionic acid, 25 g	Sigma	153516
Sucrose, 1000 g	Sigma	S0389
Bovine serum albumin, 100 g	Sigma	A7030
EGTA, 10 g	Roth	3054.1
Bradford Protein Assay, 450 mL	Bio‐Rad	500‐0006
Immonilon^®^ Western, HRP Substrate	Millipore	WBKLS0500
Pierce BCA Protein Assay Kit	Thermo	23227
Annexin V/PI Apoptosis Detect. Kit	BD‐Biosciences	556547

	Company	Cat. number	Host	Dilution
Primary antibodies				
hFAHD1 (polyclonal)	FPLC purified from rabbit serum	–	Rabbit	1 : 1000
ß‐Actin	Sigma	A5441	Mouse	1 : 20 000
α‐tubuline	Sigma	T5168	Mouse	1 : 10 000
GLS (KGA/GAC), polyclonal	Proteintech	12 855‐1‐AP	Rabbit	1 : 1000
Complex I (*NDUFA9)*	Invitrogen	459 100	Mouse	1 : 2000
Complex II (SDH)	Invitrogen	459 200	Mouse	1 : 20 000
mPEPCK	Santa Cruz	sc‐32 879	Rabbit	1 : 2000
GAPDH	Proteintech	10 494‐1‐AP	Rabbit	1 : 5000
Secondary antibodies				
Polyclonal swine‐anti‐rabbit	DAKO	P0399	Swine	1 : 3000
Polyclonal goat‐anti‐mouse	DAKO	P0447	Goat	1 : 20 000

### Statistical analysis

Experiments performed in the Oxygraph‐2k were analysed with *DatLab7*, *Seahorse* measurements were evaluated using the agilent xf Software *Seahorse Analytics* (Agilent). Data were then imported into *Microsoft Excel* and organized. Graphical visualization was mainly realized using graphpad prism v (GraphPad Software, San Diego, CA, USA) and statistical analysis was performed by comparing FAHD1 KD with SCR controls by an unpaired Student's *t*‐test, two‐tailed. A one‐way ANOVA was used for comparisons between multiple cell lines. Significant thresholds were *P ≤ 0.05; **P ≤ 0.01; ***P ≤ 0.001.

## Results

### Expression patterns of metabolic enzymes including FAHD1 differ between breast cancer subtypes

**Fig. 1:**
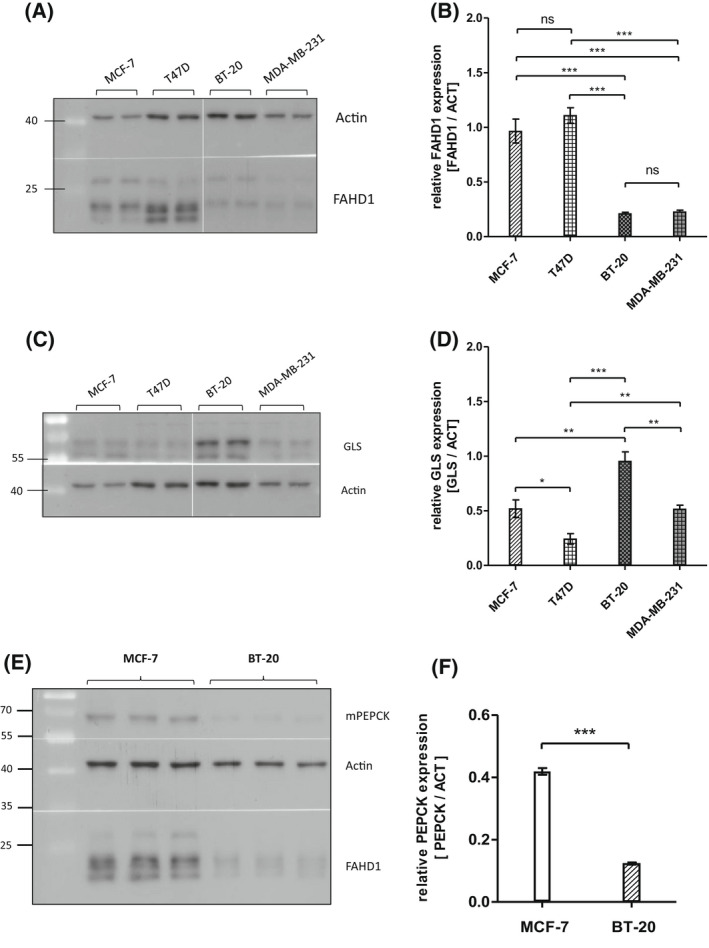
Metabolic features of different breast cancer cell lines. (A) Expression patterns of FAHD1 in various wild‐type breast cancer cell lines. (B) Evaluation of FAHD1 expression by densitometry; bars show arithmetic mean and SEM of *n* = 4 biological replicates. (C) Expression of glutaminase in various wild‐type breast cancer cell lines. (D) Densitometry of glutaminase levels; bars show arithmetic mean and SEM of *n* = 4 biological replicates. (E) Mitochondrial PEPCK levels in comparison between MCF‐7 and BT‐20; bars show arithmetic mean and SEM of *n* = 3 biological replicates. (F) Densitometry of mPEPCK levels in MCF‐7 and BT‐20 breast cancer cells, bars show arithmetic mean and SEM of *n* = 3 biological replicates. Significance threshold for Student's *t*‐test and one‐way ANOVA: *P ≤ 0.05; **P ≤ 0.01; ***P ≤ 0.001.

Immuno‐histochemical staining revealed elevated levels of FAHD1 in 75% of cells in malignant tissue derived from human breast cancer patients (Fig. S1A). Based on these findings, we investigated the expression of FAHD1 in a selection of both luminal and basal breast cancer cell lines, including MCF‐7, T47D, BT‐20 and MDA‐MB‐231. Interestingly, luminal cell lines like MCF‐7 and T47D contained higher protein levels of FAHD1 compared to basal cells lines like BT‐20 and MDA‐MB‐231 which expressed lower levels of FAHD1 (Fig. 1A,B). To get a first impression of glutaminolytic capacity of these breast cancer cell lines, expression of glutaminase (GLS) was investigated. GLS was detectable in all breast cancer cells analysed, displaying variable protein levels across our cell line panel, with the highest expression in BT‐20 cells (Fig. 1C,D). As FAHD1 is known to decarboxylate OAA, we also assessed expression levels of mitochondrial phosphoenol‐pyrvate‐carboxykinase (mPEPCK), which is an alternative step in the usage of OAA for gluconeogenesis. Of note, MCF‐7 cells express higher levels of mPEPCK compared with the other breast cancer cell lines (Fig. S1B). Direct comparison revealed significantly lower levels of mPEPCK in BT‐20 relative to MCF‐7 cells (Fig. 1E,F). For in‐depth functional analysis, we selected MCF‐7 and BT‐20 cells, representing the luminal and basal breast cancer type respectively. To address a potential role of FAHD1 in the metabolic wiring of human breast cancer cells, we aimed to generate FAHD1 KD models in luminal MCF‐7 and basal BT‐20 cells.

### Establishment of FAHD1‐deficient MCF‐7 clones

**Fig. 2:**
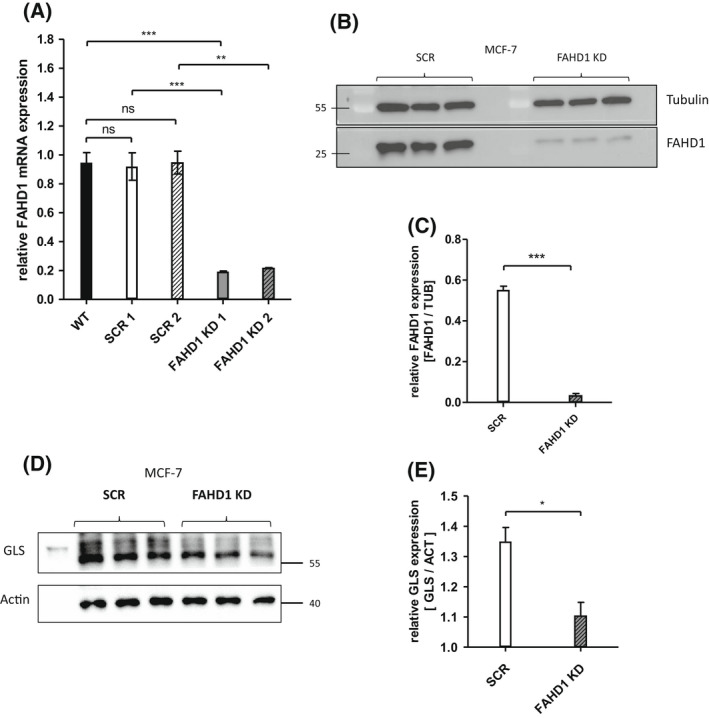
Establishment of FAHD1‐deficient MCF‐7 clones. (A) Expression of FAHD1 was analysed on mRNA and (B) protein levels, both verifying the specific knock‐down in MCF‐7. (C) Densitometry of FAHD1 expression in MCF‐7. Bars depict arithmetic mean and SEM of *n* = 3 biological replicas. (D) Relative protein levels of glutaminase (GLS) were analysed in MCF‐7 upon FAHD1 KD and were evaluated *via* (E) densitometry in imagej, *n* = 3 biological replicates with arithmetic mean and SEM. Significance threshold for Student's *t*‐test: *P ≤ 0.05; **P ≤ 0.01; ***P ≤ 0.001.

In a first attempt, the expression of FAHD1 was targeted in MCF‐7 cells by using a lentiviral RNA‐interference approach. Control cells, which were infected with untargeted scrambled siRNA (SCR), still express physiological levels of FAHD1 compared to uninfected wild‐type cells (WT, Fig. S1C). Protein and mRNA levels were strongly reduced in FAHD1 KD MCF‐7 cells (Fig. 2A–C). We also noted a significant down‐regulation of GLS expression in FAHD1‐depleted MCF‐7 cells (Fig. 2D,E). MCF‐7 FAHD1 KD cells were successfully expanded in regular DMEM, frozen in aliquots and cultures established from frozen aliquots, which, when grown in regular DMEM, showed no detectable alterations in cell growth after thawing (data not shown, see also below), indicating that depletion of FAHD1 does not significantly affect viability of MCF‐7 cells in full media.

### Depletion of FAHD1 reduces proliferation of MCF‐7 cells with glutamine as the only available carbon source

**Fig. 3:**
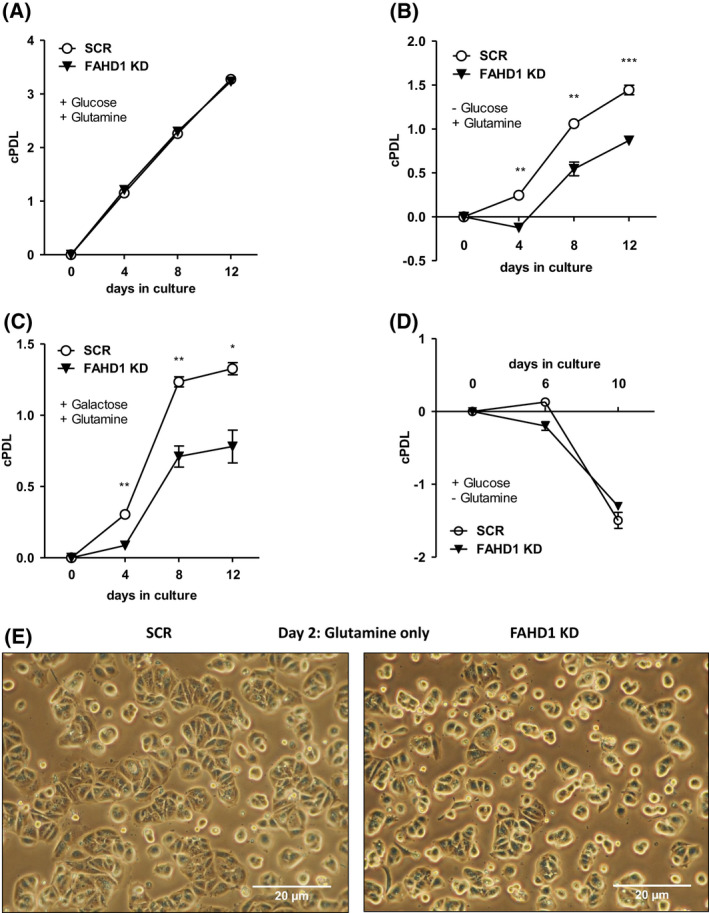
Proliferation of luminal FAHD1 KD MCF‐7. (A) Control (SCR) and FAHD1 KD cells were grown in *full media* containing glucose and glutamine; error bars show SEM of *n* = 3 biological replicates. (B) Growth impairment in MCF‐7 grown in *glutamine only* media without supplementation of glucose using dialysed serum upon FAHD1 KD and (C) cultivation of MCF‐7 cells in *glutamine only* media supplemented with galactose; error bars show SEM of *n* = 3 biological replicates. (D) MCF‐7 cultivated in *glucose only* media; error bars show SEM of *n* = 3 biological replicates. (E) Bright field microscopy of MCF‐7 cells cultivated in *glutamine only* media after 48 h under limiting conditions. Significance threshold for Student's *t*‐test: *P ≤ 0.05; **P ≤ 0.01; ***P ≤ 0.001.

The successful establishment of MCF‐7 FAHD1 KD cells allowed a detailed characterization of metabolic changes caused by depletion of FAHD1. MCF‐7 FAHD1 KD cells and controls were grown in media containing a combination of different carbon sources such as glucose, glutamine and galactose. To remove any traces of other carbon sources in limiting media conditions, dialysed FCS was used throughout to supplement the culture media. In *full media* containing standard concentrations of glucose (15 mm) and glutamine (4 mm), no difference in proliferative capacity was observed between SCR and FAHD1 KD cells, both of which performed roughly 3.5 cumulative population doublings (cPDL) within 12 days in culture (Fig. 3A). Once glucose was left out and only glutamine (4 mm) was supplemented, proliferation capacity of control (SCR) cells was reduced to roughly 1.5 cPDL in 12 days, whereas the proliferation capacity of FAHD1 KD cells was significantly further reduced (to roughly 1 cPDL in 12 days) relative to controls (Fig. 3B). Addition of galactose (15 mm) to media containing glutamine (4 mm) did not have any additional effect (Fig. 3C). Cells grown in media containing only glucose as carbon source displayed negative cPDL indicating cell death irrespective of their FAHD1 status (Fig. 3D), suggesting a strict dependency of MCF‐7 cells on glutamine. We found that growing MCF‐7 cells under limiting conditions, such as *glutamine only* or *glucose only*, led to upregulation of mPEPCK in both SCR and FAHD1 KD cells (Fig. S2A), which may be triggered by reduced availability of the respective carbon source. While no morphological alterations were observed in FAHD1 KD cells when grown in *full media* (Fig. S2B), the removal of glucose resulted in cellular stress with more misshaped and roundish cells selectively in FAHD1 KD cells (Fig. 3E).

### FAHD1 KD results in lower complex II activity and enhanced glycolytic flux

**Fig. 4:**
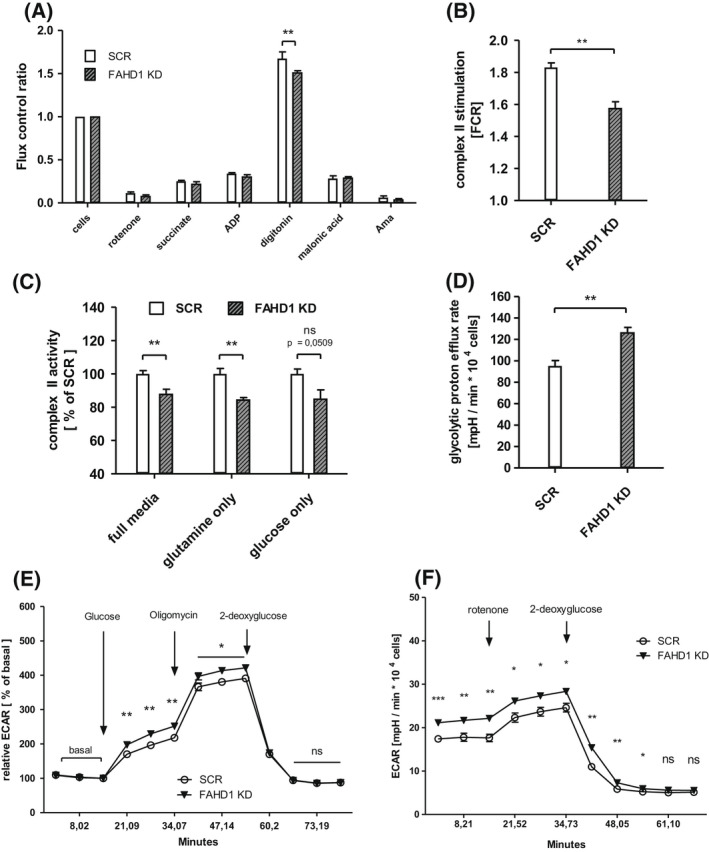
Mitochondrial function and glycolytic flux in luminal MCF‐7. (A) Respiratory rates expressed as flux control ratios in MCF‐7 cultivated in *glutamine only* media. (B) Combined complex II activities obtained from *n* = 6 biological replicates with MCF‐7 in *full media* in the *Oxygraph‐2k* with SEM. (C) Relative complex II activities in MCF‐7 cells grown in *full media*, *glutamine only* media, and *glucose only* media; *n* = 6 for *full media*, *n* = 4 for *glutamine only* and *glucose only*, respectively, with SEM. (D) Glycolytic activity based on proton efflux rates in MCF‐7 cultivated in *full media*, *n* = 6 biological replicates with SEM. (E) Relative extracellular acidification rates (ECAR) measured during the *glycolytic stress test* and (F) during the *glycolytic rate assay* in the *Seahorse XF HS Mini; n* = 6 biological replicates with SEM. Significance threshold for Students' *t*‐test: *P ≤ 0.05; **P ≤ 0.01; ***P ≤ 0.001.

Succinate dehydrogenase (SDH) is competitively inhibited by oxaloacetate which was described already 1967 by Zeylemaker et al. [24]. Previous studies identified FAHD1 as an ODx and showed accumulation of OAA in liver and kidney tissue from Fahd1‐KO mice [18]. To test succinate usage based on mitochondrial complex‐II (SDH) activity, MCF‐7 cells were loaded into an *Oxygraph‐2k* (O2K) respirometer which measures changes in oxygen consumption. The protocol chosen for this approach consists of several substrate/inhibitor titrations which allows to estimate complex II activity by: (a) blocking complex I with rotenone, (b) providing ADP and the complex‐II substrate succinate and (c) permeabilize the cells with digitonin followed by (d) inhibition of SDH with malonic acid (details in Material and methods, *High resolution respirometry*, Fig. 4A, Fig. S3A [25]). Complex II activity, indicated by increased OCR after cell permeabilization under these conditions, was significantly (by 10–15%) reduced in FAHD1 KD MCF‐7 relative to controls, when cells were either grown in *full media* (Fig. 4B, Fig. S3B) or in *glutamine only* media (Fig. 4C). In *glucose only* conditions, complex II activity was also reduced in FAHD1 KD cells relative to control although the difference did not reach statistical significance (Fig. 4C). Activities are depicted as *flux control ratios* which are internally normalized values and show the changes in oxygen consumption relative to initial basal respiration (*cells* = 1.0, Fig. 4A). In order to identify potential variations in mitochondrial content, the expression of mitochondrial complex I and II was analysed on protein levels (Fig. S2C,D). No differences were observed between SCR and FAHD1 KD MCF‐7 cells indicating that mitochondrial levels are comparable. These results provide evidence that succinate usage *via* SDH is attenuated in FAHD1 KD cells.

To test the usage of glucose in FAHD1 KD cells and controls, relative extracellular acidification rates (ECAR) based on glycolytic flux and lactate secretion, were analysed in the *Seahorse XF HS Mini*. Stressing the cells for 1 h in glucose‐free DMEM resulted in an enhanced response to reintroduced glucose in FAHD1 KD cells (Fig. 4E) as part of the *glycolytic stress test*. The addition of oligomycin (an inhibitor of complex V/ATP‐Synthase) stimulated glycolytic flux, which was subsequently blocked by the addition of the hexokinase inhibitor 2‐deoxy‐glucose (2‐DG). Absolute ECAR induction by glucose was also significantly higher in FAHD1 KD cells (Fig. S3C), suggesting increased glycolytic metabolism in the absence of FAHD1. This was further confirmed by a *glycolytic rate assay* which showed higher proton efflux rate and a higher overall ECAR in FAHD1 KD MCF‐7 (Fig. 4D,F). Similar to the results obtained with the *glycolytic stress test*, 2‐DG fully blocked ECAR signals verifying that mainly glycolysis is contributing to extracellular acidification detected in the *Seahorse XF HS Mini*.

Taken together, these experiments reveal a partial shift from mitochondrial metabolism towards increased glycolytic flux in FAHD1 KD cells.

### FAHD1 depletion is not compatible with survival of BT‐20 cells

**Fig. 5:**
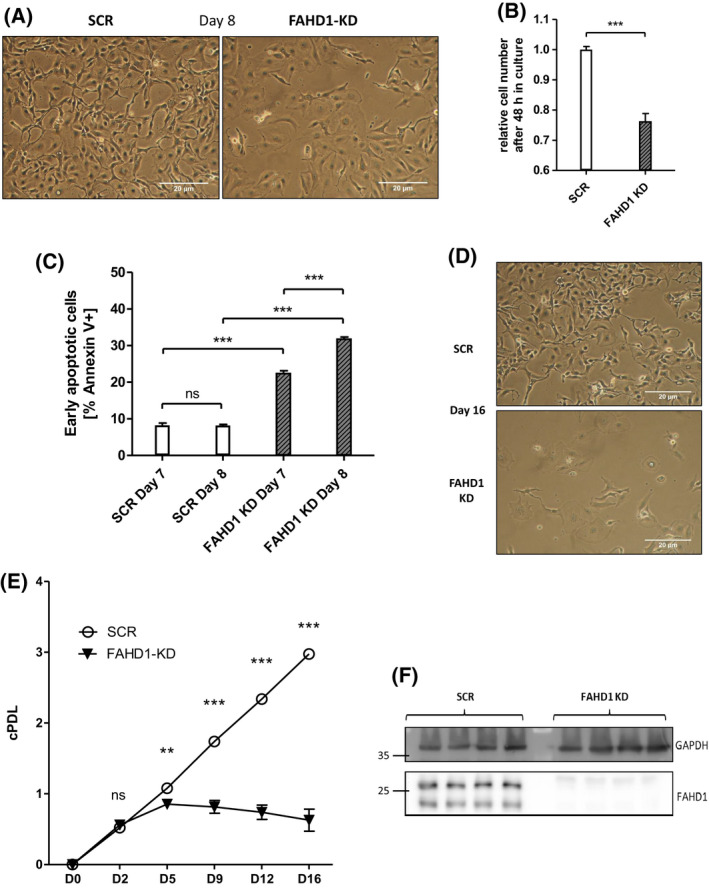
FAHD1 KD causes lower proliferation and cell death in basal BT‐20. (A) Bright field pictures of BT‐20 at day 8 post infection (10 MOI). (B) Cell numbers were monitored between day 7 and day 9 (48 h) post infection with 10 MOI; *n* = 5 biological replicates with SEM (C) Early apoptosis (Annexin V+) was detected by flow‐cytometry at day 7 and day 8 post infection; *n* = 3 biological replicates with SEM. (D) Bright field pictures showing collapsed BT‐20 culture upon FAHD1 KD at day 16 post infection. (E) Growth curve of control (SCR) and FAHD1 KD BT‐20 cells over 16 days in culture; *n* = 3 biological replicates with SEM. (F) Western blot showing depleted FAHD1 expression on protein levels in BT‐20 FAHD1 KD cells. Significance threshold for Student's *t*‐test: *P ≤ 0.05; **P ≤ 0.01; ***P ≤ 0.001.

Similar to experiments described for MCF‐7 cells, BT‐20 cells were infected with lentiviral vectors carrying a shRNA targeting FAHD1, as described above, cultured under puromycin selection, and monitored for cell proliferation. In contrast to MCF‐7 cells, where stable FAHD1 KD clones could be easily obtained (see above), FAHD1 KD in BT‐20 cells induced severe morphological alterations after a few days in culture (Fig. 5A). Moreover, total cell numbers declined over time (Fig. 5B), involving the activation of programmed cell death (Fig. 5C, Fig. S4A), as detected by Annexin V flow‐cytometry. As of day 5, cell numbers started to decrease (Fig. 5D,E). To be able to collect information on the metabolic fate of FAHD1‐depleted BT‐20 cells, we decided to characterize metabolic consequences of FAHD1 KD in early passages (day 5 post‐infection) of freshly infected BT‐20 cells.

Protein lysates were obtained from cells on day 5 post‐infection, when cell numbers were still equivalent between FAHD1 KD cells and controls, and analysed for levels of FAHD1 and GLS. Similar to the results obtained with MCF‐7, transient infection of BT‐20 cells with a lentiviral vector carrying a shRNA targeting FAHD1, reduced FAHD1 expression relative to control cells (Fig. 5F). Cell numbers continued to decrease with extended passaging and resulted in the collapse of FAHD1 KD cultures (Fig. 5D,E).

These findings indicate that it was not possible to establish stable clones of FAHD1 KD BT‐20 cells, suggesting that FAHD1 is essential for the proliferation and/or viability of BT‐20 cells.

### Initial metabolic characterization of FAHD1‐depleted BT‐20 cells

**Fig. 6:**
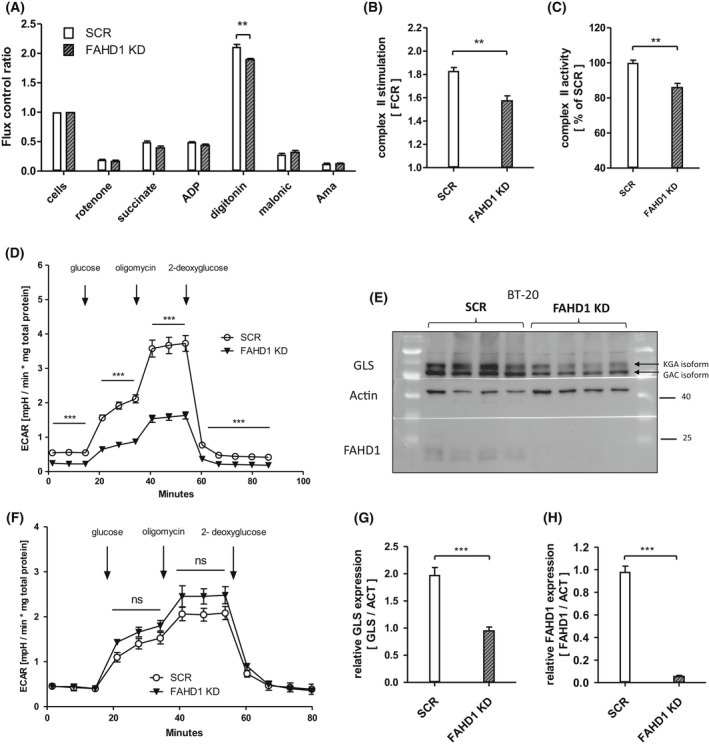
Complex II deficiency and lower GLS levels in FAHD1 KD cells. (A) Respiratory rates expressed as flux control ratios in BT‐20 at day 5 post infection (B) Evaluation of summarized complex II activity expressed as *flux control ratio* (FCR) and (C) relative complex II activity depicted as *per cent* (%) of control (SCR); *n* = 4 biological replicates with SEM. (D) *Glycolytic stress test* with BT‐20 at day 8 post infection; *n* = 6 with SEM. (E) Western blot showing GLS expression in control and FAHD1 KD BT‐20 cells at day 10 post infection (F) *Glycolytic stress test* with MDA‐MB‐231; *n* = 3 with SEM. (G) Evaluation of overall GLS expression and (H) FAHD1 expression in BT‐20 by densitometry; *n* = 8 biological replicates with SEM. Significance threshold for Student's *t*‐test: *P ≤ 0.05; **P ≤ 0.01; ***P ≤ 0.001.

Similar to the experiments performed with MCF‐7, complex II function was analysed in BT‐20 (SCR vs. FAHD1 KD) cells using the *Oxygraph‐2K*. Consistently, succinate usage by SDH was significantly (by 15%) reduced in FAHD1 KD cells (Fig. 6A–C) confirming the findings in luminal MCF‐7. This indicates that the activity of FAHD1 influences the TCA cycle flux through complex‐II and favours the conversion of succinate. In contrast to results obtained with MCF‐7 cells (see above), glycolytic flux analysed in the *Seahorse XF HS Mini* was significantly reduced by FAHD1 KD in BT‐20 cells (Fig. 6D). To compare this result with another triple‐receptor‐negative breast cancer cell line, glycolytic flux was measured in MDA‐MB‐231 cells during a *glycolytic stress test* and a *glycolytic rate assay*. FAHD1 KD MDA‐MB‐231 cells show increased ECAR (commonly associated with glycolytic flux) when glucose is reintroduced during the *glycolytic stress test* (Fig. 6F) while basal glycolytic rates were not altered between SCR and FAHD1 cells (Fig. S3D).

When GLS levels were analysed in BT‐20 cells we found that both GLS isoforms, namely KGA and GAC, were downregulated roughly two‐fold upon FAHD1 KD in BT‐20 cells (Fig. 6E,G, Fig. S4B,C). FAHD1 expression was at < 10% compared to control cells (Fig. 6E,H). Unlike in MCF‐7 (Fig. S2A), limiting the availability of either glucose or glutamine in the culture medium did not stimulate mPEPCK expression in BT‐20 (Fig. S4D). No differences in mPEPCK expression were observed between SCR and FAHD1 KD BT‐20. Mitochondrial contents were not altered substantially between SCR and FAHD1 KD cells as indicated by protein levels of mitochondrial complex I and complex II (Fig. S4E).

In summary, the depletion of FAHD1 led to lower complex‐II activity in BT‐20 cells accompanied by metabolic alterations such as lower glycolytic flux and down‐regulation of GLS levels which resulted in cell death.

## Discussion

In this study, we described the role of the mitochondrial oxaloacetate decarboxylase FAHD1 in breast cancer. As a member of the basal breast cancer subtypes, BT‐20 cells represent a distinct group of highly glutamine affine cells, including basal breast cancer cell lines H1143, H1569 and H1500 [26]. Effects of FAHD1 withdrawal were more severe in BT‐20 cells compared to luminal MCF‐7. The expression of certain metabolic enzymes [27], for example mPEPCK, differs strongly between luminal and basal subtypes. While MCF‐7 cells are able to shuttle OAA from the mitochondria to the cytosol and restore PEP and pyruvate levels under stressful conditions (Fig. S2A), this pathway appears to be inactive in BT‐20 cells when substrates are limited (Fig. 1E,F; Fig. S4D). The subsequent accumulation of OAA, due to the depletion of FAHD1 from the mitochondrial environment, leads to a lower complex‐II activity. Due to the impaired conversion of succinate by SDH in FAHD1 KD cells, the TCA cycle flux is interrupted. The usage of glutamine (*via* GLS*)* to fuel the TCA cycle is less efficient as α‐ketoglutaric acid cannot be converted further to yield malate or oxaloacetate, leading to an unfavourable metabolic situation. This increases glycolytic flux in luminal MCF‐7 and to some extend in basal MDA‐MB‐231 in order to compensate for impaired mitochondrial function (lower SDH activity). Recently, similar compensatory mechanisms were described in a murine FAHD1‐KO model when cellular reprogramming was induced [28]. One key finding reported in the present study is the unexpected decrease of GLS protein levels in FAHD1‐depleted breast cancer cells, which may have widespread consequences for the metabolic state of these cells. Decreased expression of GLS can reduce glutaminolysis, diminishing the ability to synthesize macromolecules and impairing cell growth and proliferation, especially in cancer. The conversion of glutamine to glutamate performed by GLS determines intracellular glutamate levels and can therefore impact other homeostatic reactions [7]. Glutamate has multiple roles as donor of amino‐groups in anaplerotic reactions throughout the cell. As a substrate of the malate–aspartate shuttle, it enables the transport of OAA (in the form of aspartate) through the mitochondrial membrane. Similarly, glutamate provides the nitrogen for the molecule phosphoserine, a precursor for serine‐biosynthesis [29, 30]. In this process, the cofactor pyridoxal‐5‐phosphate plays a crucial role which is synthetized partially from glutamate and pyruvate. Serine can then be integrated in the sphingolipid metabolism which was shown to be important in cancer cell proliferation [31], ROS signalling [32, 33] and development of chemoresistance [34]. Also, the import of cystine by the antiporter xCT (SLC7A11) is mediated by the secretion of glutamate and represents an important step for glutathione synthesis [35, 36]. It was shown that this process is relevant in breast cancer cells to maintain redox balance and counteract high mitochondrial activity coupled to the production of reactive oxygen species [37]. The reduction of cystine to cysteine is coupled to NADPH consumption which is provided by the pentose‐phosphate‐pathway and was shown to be connected to glucose import [38]. In a model derived from these findings, the availability of glutamate should be limited due to lower GLS levels. The import of glutamine [3], GLS activity [12] and glutamate availability for the import of cystine *via* xCT [26] was shown to be central for TNBC in order to tolerate a highly oxidative environment [39]. This defence system protects cancer cells from eventually entering necroptosis and ferroptosis [40] and to maintain the intracellular redox balance [41, 42]. The molecular cascade by which FAHD1 withdrawal activates cell death in BT‐20 cells (Fig. 5C–E) will be subject to future studies. The apparent inability to shift the metabolism towards glycolysis in response to FAHD1 KD may contribute to cellular stress in BT‐20 and eventually lead to apoptosis. We found that ECAR is lower in MCF‐7 compared to BT‐20, with MDA‐MB‐231 cells somewhat ‘in between’, which is in accordance with their phenotype. To some extent such variability between breast cancer cell subtype types is supported by extensive literature on subtle metabolic differences between breast cancer subtypes. While MCF‐7 cells can survive FAHD1 depletion, BT‐20 cells do not tolerate a FAHD1 knockdown. MDA‐MB‐231 cells are apparently stressed upon FAHD1 knockdown, but survive under normal conditions. In summary, the data are consistent with the commonly held view that individual patterns of metabolic rewiring are a common feature of established cancer cell lines, which still are very important models in cancer research. Our current findings provide new insights to the field of cancer metabolism and could enable new therapeutic strategies for tumours that are relying on glutamine as a fast energy source for mitochondrial metabolism.

## Author contributions

Experiments were conducted and analysed by MH with the help of LG, ED, EA, AS and MP in establishing the cell lines which were provided by HF and WP. IHC experiments were performed by FS using the α‐hFAHD1‐antibody described in 2015 by Pircher et al. The study and manuscript were conceptualized by MH under the supervision of PJ and AW.

## Supporting information


**Fig. S1.** Expression of FAHD1 in breast cancer tissue and verification of regular FAHD1 levels in control cells.
**Fig. S2.** MCF‐7 cells cultivated in limiting conditions and in full media.
**Fig. S3.** High resolution respirometry for complex II activity.
**Fig. S4.** Part 2. Cell death and expression of GLS in FAHD1 KD BT‐20 cells.

## Data Availability

The data supporting the findings of this study are available within the article and its supplementary materials. Additional data that support the findings of this study are available from the corresponding author upon reasonable request.

## References

[1] Grasso D , Zampieri LX , Capelôa T , Van de Velde JA , Sonveaux P . Mitochondria in cancer. Cell Stress. 2016;4(6):114–1. 10.15698/cst2020.06.221 PMC7278520 32548570

[2] Warburg O . [On the facultative anaerobiosis of cancer cells and its use in chemotherapy]. *Münch Med Wochenschr*. 1950;103:2504–6. 14004908

[3] Morotti M , Zois CE , El‐Ansari R , Craze ML , Rakha EA , Fan SJ , et al. Increased expression of glutamine transporter SNAT2/SLC38A2 promotes glutamine dependence and oxidative stress resistance, and is associated with worse prognosis in triple‐negative breast cancer. Br J Cancer. 2021;124(2):494–505. 33028955 10.1038/s41416-020-01113-y PMC7852531

[4] Altman BJ , Stine ZE , Dang CV . From Krebs to clinic: glutamine metabolism to cancer therapy. Nat Rev Cancer. 2016;16(10):619–34. 27492215 10.1038/nrc.2016.71 PMC5484415

[5] Hensley CT , Wasti AT , DeBerardinis RJ . Glutamine and cancer: cell biology, physiology, and clinical opportunities. J Clin Invest. 2013;123(9):3678–84. 23999442 10.1172/JCI69600 PMC3754270

[6] DeBerardinis RJ , Mancuso A , Daikhin E , Nissim I , Yudkoff M , Wehrli S , et al. Beyond aerobic glycolysis: transformed cells can engage in glutamine metabolism that exceeds the requirement for protein and nucleotide synthesis. Proc Natl Acad Sci USA. 2007;104(49):19345–50. 18032601 10.1073/pnas.0709747104 PMC2148292

[7] Sharma MK , Seidlitz EP , Singh G . Cancer cells release glutamate via the cystine/glutamate antiporter. Biochem Biophys Res Commun. 2010;391(1):91–5. 19896463 10.1016/j.bbrc.2009.10.168

[8] Carey BW , LWS F , Cross JR , Allis CD , Craig B . Intracellular α‐ketoglutarate maintains the pluripotency of embryonic stem cells. Nature. 2015;518:413–6. 25487152 10.1038/nature13981 PMC4336218

[9] Tran KA , Dillingham CM , Sridharan R . The role of α‐ketoglutarate dependent proteins in pluripotency acquisition and maintenance. J Biol Chem. 2019;294(14):5408–19. 30181211 10.1074/jbc.TM118.000831 PMC6462505

[10] Cheishvili D , Boureau L , Szyf M . DNA demethylation and invasive cancer: implications for therapeutics. Br J Pharmacol. 2015;172(11):2705–15. 25134627 10.1111/bph.12885 PMC4439869

[11] Tran TQ , Hanse EA , Habowski AN , Li H , Gabra MBI , Yang Y , et al. α‐Ketoglutarate attenuates Wnt signaling and drives differentiation in colorectal cancer. Nat Cancer. 2020;1(3):345–3. 32832918 10.1038/s43018-020-0035-5 PMC7442208

[12] Lampa M , Arlt H , He T , Ospina B , Reeves J , Zhang B , et al. Glutaminase is essential for the growth of triple‐negative breast cancer cells with a deregulated glutamine metabolism pathway and its suppression synergizes with mTOR inhibition. PLoS One. 2017;12(9):e0185092. 28950000 10.1371/journal.pone.0185092 PMC5614427

[13] Dos Reis LM , Adamoski D , Souza ROO , Ascenção CFR , De Oliveira KRS , Corrêa‐Da‐Silva F , et al. Dual inhibition of glutaminase and carnitine palmitoyltransferase decreases growth and migration of glutaminase inhibition‐resistant triple‐negative breast cancer cells. J Biol Chem. 2019;294(24):9342–57. 31040181 10.1074/jbc.RA119.008180 PMC6579458

[14] Robinson MM , McBryant SJ , Tsukamoto T , Rojas C , Ferraris DV , Hamilton SK , et al. Novel mechanism of inhibition of rat kidney‐type glutaminase by bis‐2‐(5‐phenylacetamido‐1,2,4‐thiadiazol‐2‐yl)ethyl sulfide (BPTES). Biochem J. 2007;406(3):4. 10.1042/BJ20070039 PMC2049044 17581113

[15] Gross MI , Demo SD , Dennison JB , Chen L , Chernov‐Rogan T , Goyal B , et al. Antitumor activity of the glutaminase inhibitor CB‐839 in triple‐negative breast cancer. Mol Cancer Ther. 2014;13(4):890–901. 24523301 10.1158/1535-7163.MCT-13-0870

[16] Kung HN , Marks JR , Chi JT . Glutamine synthetase is a genetic determinant of cell type‐specific glutamine independence in breast epithelia. PLoS Genet. 2011;7(8):e1002229. 21852960 10.1371/journal.pgen.1002229 PMC3154963

[17] Bianchini G , Balko JM , Mayer IA , Sanders MEGL . Triple‐negative breast cancer: challenges and opportunities of a heterogeneous disease. Nat Rev Clin Oncol. 2016;13(11):674–90. 27184417 10.1038/nrclinonc.2016.66 PMC5461122

[18] Pircher H , Von Grafenstein S , Diener T , Metzger C , Albertini E , Taferner A , et al. Identification of FAH domain‐containing protein 1 (FAHD1) as oxaloacetate decarboxylase. J Biol Chem. 2015;290(11):6755–62. 25575590 10.1074/jbc.M114.609305 PMC4358102

[19] Etemad S , Petit M , AKH W , Schrattenholz A , Baraldo G , Jansen‐Dürr P . Oxaloacetate decarboxylase FAHD1 – a new regulator of mitochondrial function and senescence. Mech Ageing Dev. 2019;177:22–9. 30055189 10.1016/j.mad.2018.07.007

[20] Weiss AKH , Loeffler JR , Liedl KR , Gstach H , Jansen‐Dürr P . The fumarylacetoacetate hydrolase (fah) superfamily of enzymes: multifunctional enzymes from microbes to mitochondria. Biochem Soc Trans. 2018;46:295–309. 29487229 10.1042/BST20170518

[21] Petit M , Koziel R , Etemad S , Pircher H , Jansen‐Dürr P . Depletion of oxaloacetate decarboxylase FAHD1 inhibits mitochondrial electron transport and induces cellular senescence in human endothelial cells. Exp Gerontol. 2017;92:7–12. 28286170 10.1016/j.exger.2017.03.004

[22] Parson W , Kirchebner R , Mühlmann R , Renner K , Kofler A , Schmidt S , et al. Cancer cell line identification by short tandem repeat profiling: power and limitations. FASEB J. 2005;19(3):1–18. 15637111 10.1096/fj.04-3062fje

[23] Kim HR , Lee J , Byeon JS , Gu NY , Lee J , Cho IS , et al. Extensive characterization of feline intra‐abdominal adipose‐derived mesenchymal stem cells. J Vet Sci. 2017;18(3):299–306. 10.4142/jvs.2017.18.3.299 PMC5639082 27456770

[24] Zeylemaker W , Slater E . The inhibition of succinate dehydrogenase by oxaloacetate. Biochim Biophys Acta. 1967;132:212–4. 6030355 10.1016/0005-2744(67)90214-8

[25] Rötig A , Munnich A . Genetic features of mitochondrial respiratory chain disorders. J Am Soc Nephrol. 2003;14(12):2995–3007. 14638899 10.1097/01.asn.0000095481.24091.c9

[26] Timmerman LA , Holton T , Yuneva M , Louie RJ , Daemen A , Hu M , et al. Glutamine sensitivity analysis identifies the xCT antiporter as a common triple‐negative basal‐like breast cancer tumor therapeutic target. Cancer Cell. 2013;24(4):450–65. 24094812 10.1016/j.ccr.2013.08.020 PMC3931310

[27] Castegna A , Menga A . Glutamine synthetase: localization dictates outcome. Genes (Basel). 2018;9(2):108. 29463059 10.3390/genes9020108 PMC5852604

[28] Salti A , Etemad S , Cubero MS , Albertini E , Kovacs‐Szalka B , Holzknecht M , et al. High glycolytic activity enhances stem cell reprogramming of fahd1‐ko mouse embryonic fibroblasts. Cell. 2021;10(8):2040. 10.3390/cells10082040 PMC8392800 34440809

[29] Singh RK , Kumar D , Gourinath S . Phosphoserine aminotransferase has conserved active site from microbes to higher eukaryotes with minor deviations. Protein Pept Lett. 2021;28(9):996–1008. 33588715 10.2174/0929866528666210215140231

[30] Ravez S , Spillier Q , Marteau R , Feron O , Frédérick R . Challenges and opportunities in the development of serine synthetic pathway inhibitors for cancer therapy. J Med Biochem. 2017;60(4):1227–37. 10.1021/acs.jmedchem.6b01167 27959531

[31] Ponnusamy S , Meyers‐Needham M , Senkal CE , Saddoughi SA , Sentelle D , Selvam SP , et al. Sphingolipids and cancer: ceramide and sphingosine‐1‐phosphate in the regulation of cell death and drug resistance. Future Oncol. 2010;6(10):1603–24. 21062159 10.2217/fon.10.116 PMC3071292

[32] Maceyka M , Milstien S , Spiegel S . Shooting the messenger oxidative stress regulates Sphingosine‐1‐phosphate. Circ Res. 2007;100(1):7–9. 17204658 10.1161/01.RES.0000255895.19868.a3

[33] de Faria Poloni J , Chapola H , Feltes BC , Bonatto D . The importance of sphingolipids and reactive oxygen species in cardiovascular development. Biol Cell. 2014;106(6):167–81. 24678717 10.1111/boc.201400008

[34] Shammout ODA , Ashmawy NS , Shakartalla SB , Altaie AM , Semreen MH , Omar HA , et al. Comparative sphingolipidomic analysis reveals significant differences between doxorubicin‐sensitive and ‐resistance MCF‐7 cells. PLoS One. 2021;16:1–19. 10.1371/journal.pone.0258363 PMC8509934 34637456

[35] Linher‐Melville K , Haftchenary S , Gunning P , Singh G . Signal transducer and activator of transcription 3 and 5 regulate system xc‐ and redox balance in human breast cancer cells. Mol Cell Biochem. 2015;405(1–2):205–21. 25896132 10.1007/s11010-015-2412-4

[36] Lim JKM , Delaidelli A , Minaker SW , Zhang HF , Colovic M , Yang H , et al. Cystine/glutamate antiporter xCT (SLC7A11) facilitates oncogenic RAS transformation by preserving intracellular redox balance. Proc Natl Acad Sci USA. 2019;116(19):9433–42. 31000598 10.1073/pnas.1821323116 PMC6511045

[37] Lu L , Dong J , Wang L , Xia Q , Zhang D , Kim H , et al. Activation of STAT3 and Bcl‐2 and reduction of reactive oxygen species (ROS) promote radioresistance in breast cancer and overcome of radioresistance with niclosamide. Oncogene. 2018;37:5292–304. 29855616 10.1038/s41388-018-0340-y

[38] Liu X , Olszewski K , Zhang Y , Lim EW , Shi J , Zhang J , et al. Cystine transporter regulation of pentose phosphate pathway dependency and disulfide stress exposes a targetable metabolic vulnerability in cancer. Nat Cell Biol. 2020;22(4):476–86. 32231310 10.1038/s41556-020-0496-x PMC7194135

[39] Gurer‐Orhan H , Ince E , Konyar D , Saso L , Suzen S . The role of oxidative stress modulators in breast cancer. Curr Med Chem. 2018;25(33):4084–101. 28699501 10.2174/0929867324666170711114336

[40] Chen MS , Wang SF , Hsu CY , Yin PH , Yeh TS , Lee HC , et al. CHAC1 degradation of glutathione enhances cystine‐starvation induced necroptosis and ferroptosis in human triple negative breast cancer cells via the GCN2‐eIF2α‐ATF4 pathway. Oncotarget. 2017;8(70):114588–602. 29383104 10.18632/oncotarget.23055 PMC5777716

[41] Shanware NP , Mullen AR , DeBerardinis RJ , Abraham RT . Glutamine: pleiotropic roles in tumor growth and stress resistance. J Mol Med. 2011;89(3):229–36. 21301794 10.1007/s00109-011-0731-9

[42] Lora J , Alonso FI , Segura JA , Lobo C , Márquez J , Matés JM . Antisense glutaminase inhibition decreases glutathione antioxidant capacity and increases apoptosis in Ehrlich ascitic tumour cells. Eur J Biochem. 2004;271(21):4298–306. 15511236 10.1111/j.1432-1033.2004.04370.x

