# Supplementary material for: The mitochondrial enzyme FAHD1 regulates complex II activity in breast cancer cells and is indispensable for basal BT‐20 cells in vitro
Source: FEBS Lett. 2022 Aug 12;596(21):2781–94. doi: 10.1002/1873-3468.14462 (PMC7613834; doi:10.1002/1873-3468.14462)
Supplement: Supplementary file 1 — Fig. S1. Expression of FAHD1 in breast cancer tissue and verification of regular FAHD1 levels in control cells. Fig. S2. MCF‐7 cells cultivated in limiting conditions and in full media. Fig. S3. High resolution respirometry for complex II activity. Fig. S4. Part 2. Cell death and expression of GLS in FAHD1 KD BT‐20 cells. [file FEB2-596-2781-s001.pdf]

Supplementary Figure 1

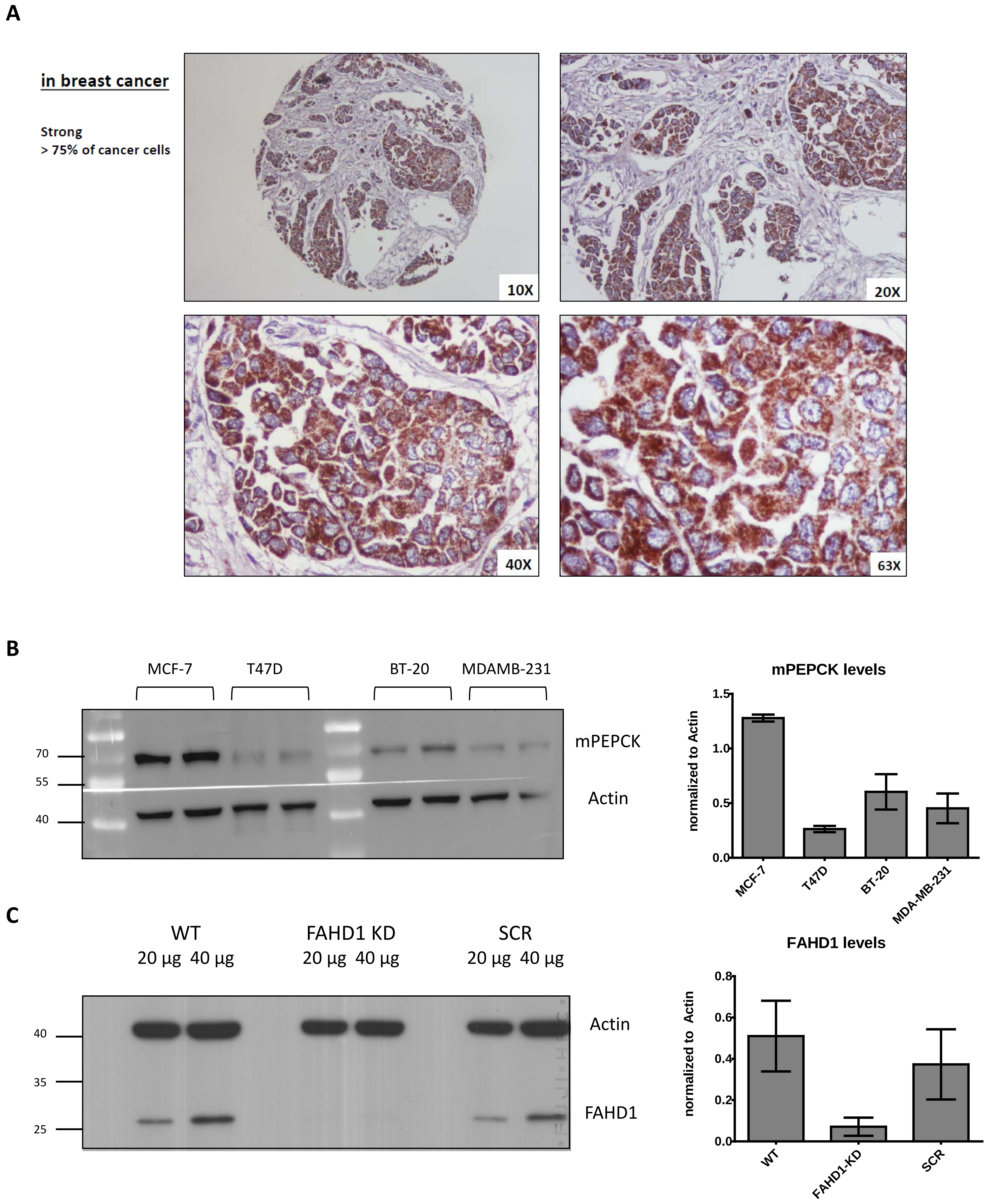

Supplementary Figure 1. Expression of FAHD1 in breast cancer tissue and verification of regular FAHD1 levels in control cells. A) The abundance of FAHD1 in breast cancer tissue from patient samples were analyzed by immuno-histochemical staining and confirmed high levels of FAHD1 in malignant tissue. **B)** Mitochondrial PEPCK expression in breast cancer cell lines analyzed by Western Blot **C)** Western blot analysis of infected MCF-7 cells shows similar FAHD1 levels in uninfected (WT) and control (SCR) cells while FAHD1 KD cells do not express the enzyme at physiological levels.

Supplementary Figure 2

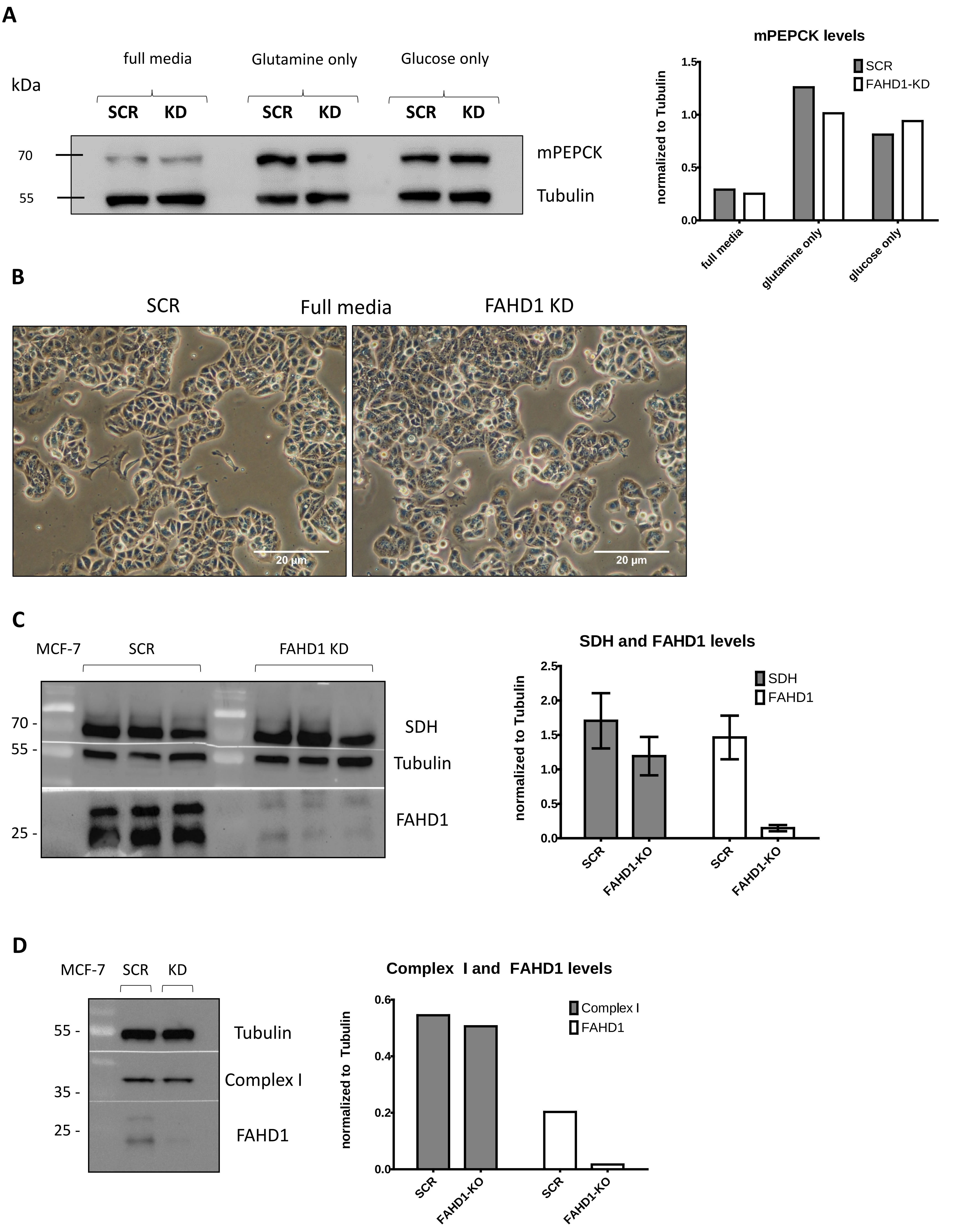

Supplementary Figure 2. MCF-7 cells cultivated in limiting conditions and in full media

A) Western blot depicting upregulation of mPEPCK in MCF-7 cells under limiting conditions such as *glutamine only* or *glucose only* media. **B)** MCF-7 cultured in regular media containing glucose and glutamine (*full media*) with no apparent morphological alterations. **C)** Protein levels of succinate dehydrogenase (SDH, mitochondrial complex II) were analyzed in control (SCR) and FAHD1 KD MCF-7 cells *via* western blot. **D)** Expression levels of NADH:ubiquinone-oxidoreductase (mitochondrial complex I, *NDUFA9*) were analyzed in SCR and FAHD1 KD MCF-7 cells.

Supplementary Figure 3

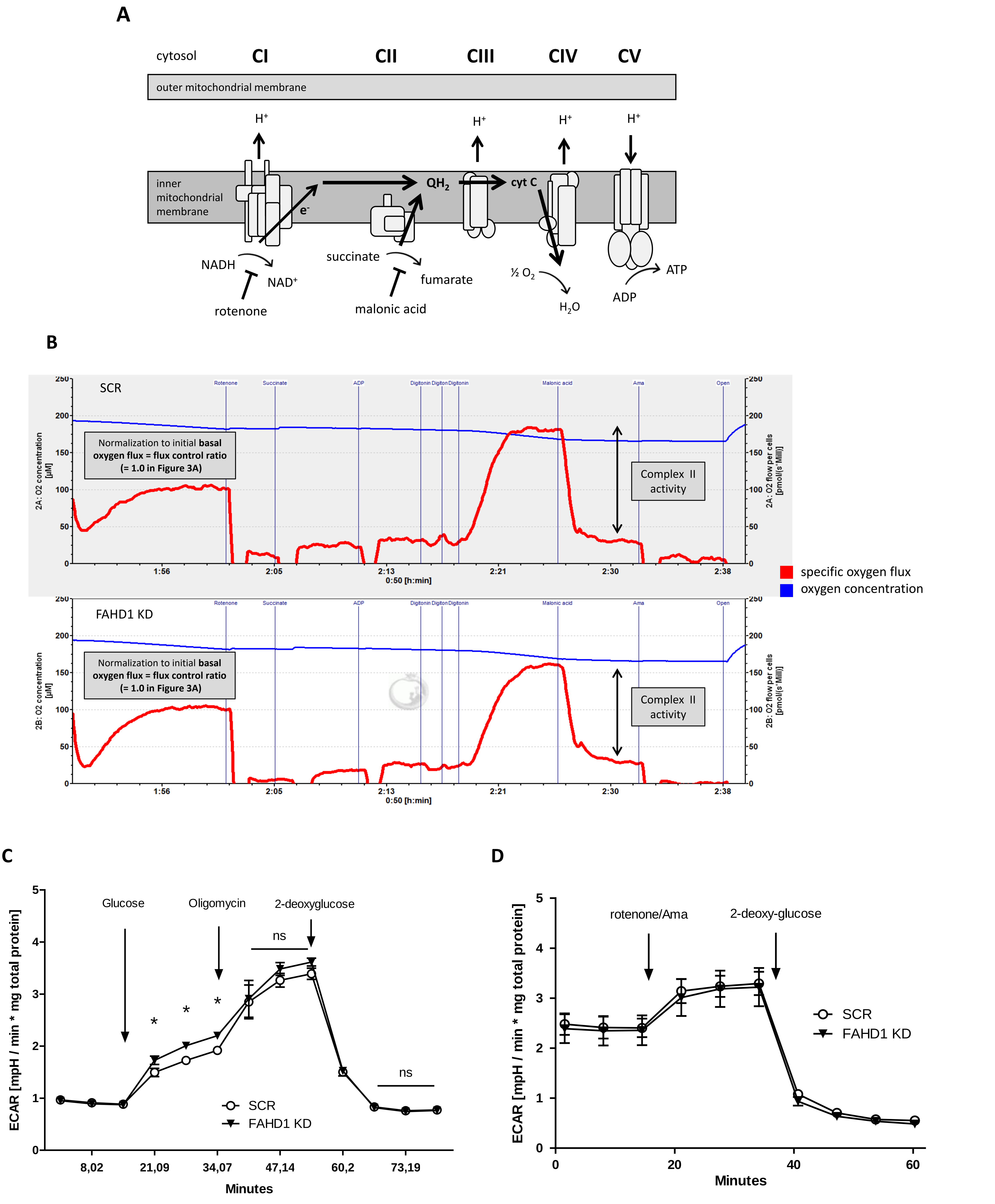

Supplementary Figure 3. High resolution respirometry for complex II activity

A) Schematic overview of the way of action for the compounds and substrates that were added during the measurement in the *Oxygraph-2k*. Details can be found at bioblast.at (SUIT-010) showing the respiratory states which were analyzed during high resolution respirometry. **B)** Representative measurement of MCF-7 breast cancer cells in *full media* showing absolute values for oxygen flux in the *Oxygraph-2k*. **C)** Absolute extracellular acidification rates (ECAR) in MCF-7 during the *glycolytic stress test* in the *Seahorse HS Mini*; n = 6 with SEM. **D)** *Glycolytic rate assay* with MDA-MB-231 cells in the *Seahorse HS Mini;* n = 3 with SEM. Significance threshold for students t-test: * $p \leq 0.05$; ** $p \leq 0.01$; *** $p \leq 0.001$

Supplementary Figure 4 – Part 1

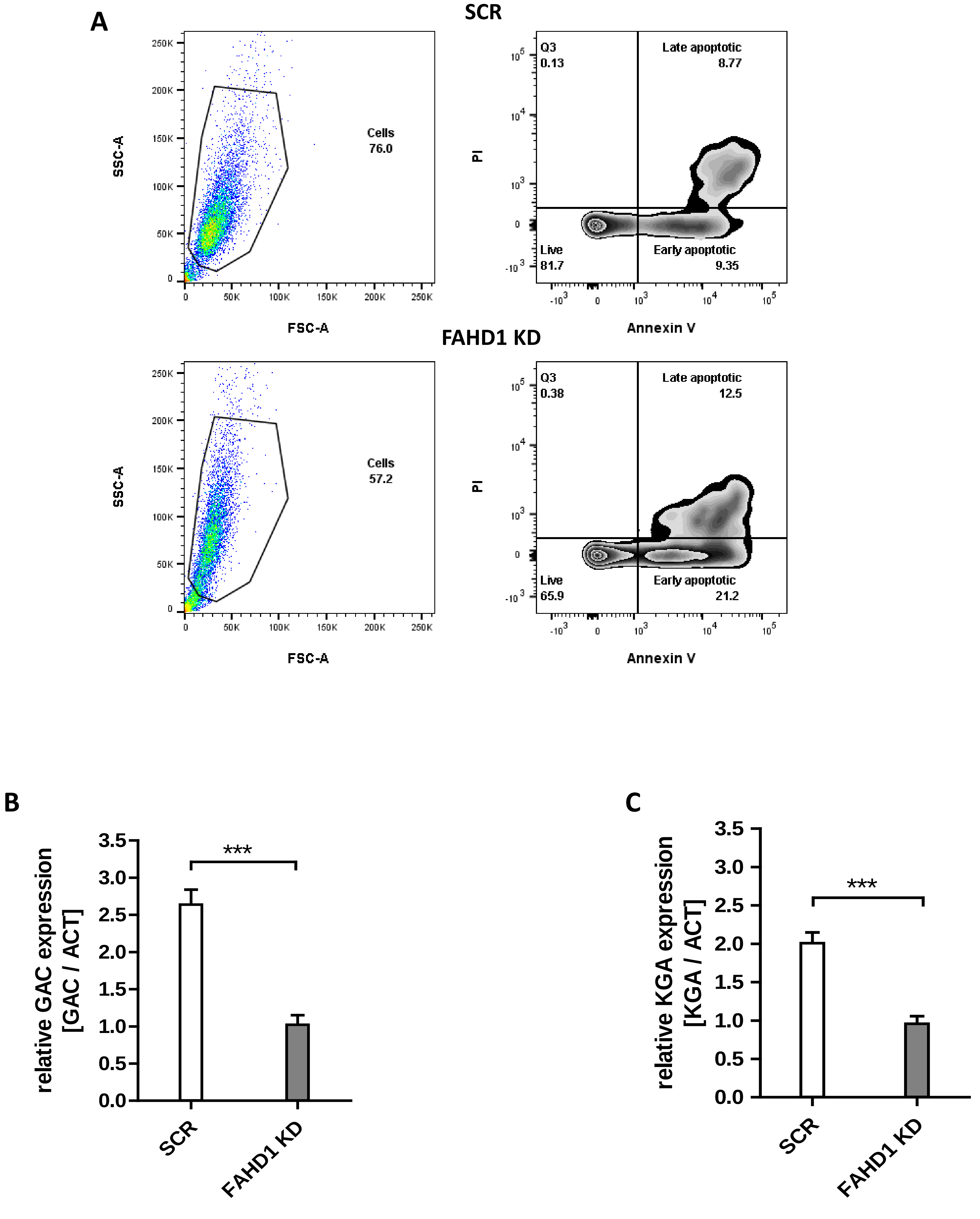

Supplementary Figure 4 – Part 1. Cell death and expression of GLS in FAHD1 KD BT-20 cells
A) Gating strategy for flow-cytometric evaluation of apoptosis in basal BT-20 cells. Representative figure for day 7 post infection. Percentages of Annexin V+ cells (early apoptotic) from the living cell population (SSC-A/FSC-A) where obtained from n = 3 biological replicates of each control (SCR) and FAHD1 KD cells. **B)** Evaluation of GLS isoform GAC expression and **C)** KGA expression in BT-20 cells upon FAHD1 KD. Mean of n = 8 biological replicates with SEM, also see Figure 5E. **D)** Levels of mitochondrial phosphoenolpyruvate-carboxykinase (mPEPCK) were measured *via* western blot in SCR and FAHD1 KD BT-20 cells which were culture for 48 h in limiting conditions. **E)** Protein expression of succinate-dehydrogenase (Complex II, SDH) and NADH:ubiquinone-oxidoreductase (mitochondrial complex I, *NDUFA9*) were analyzed in SCR and FAHD1 KD BT-20 cells. Significance threshold for students t-test: * p ≤ 0.05; ** p ≤ 0.01; *** p ≤ 0.001

Supplementary Figure 4 – Part 2

D

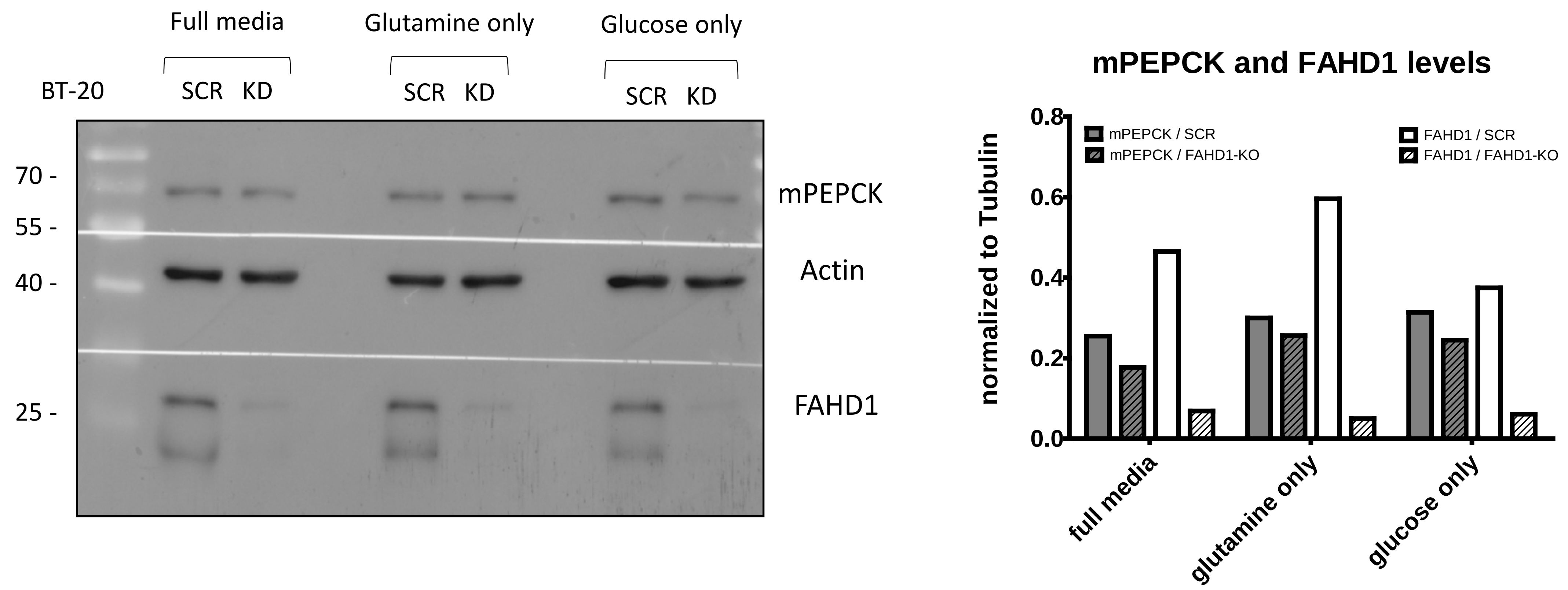

E

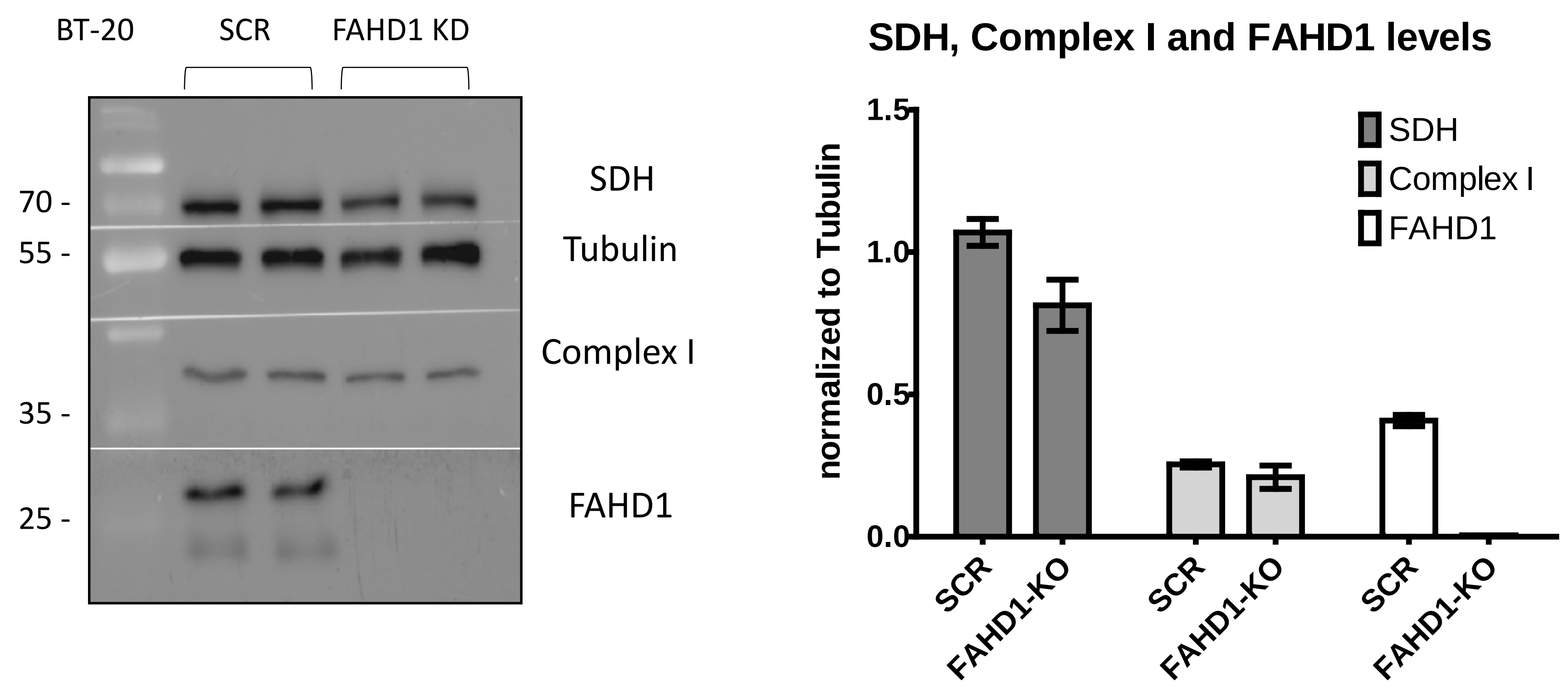

Supplementary Figure 4 – Part 2. Cell death and expression of GLS in FAHD1 KD BT-20 cells
A) Gating strategy for flow-cytometric evaluation of apoptosis in basal BT-20 cells. Representative figure for day 7 post infection. Percentages of Annexin V+ cells (early apoptotic) from the living cell population (SSC-A/FSC-A) where obtained from n = 3 biological replicates of each control (SCR) and FAHD1 KD cells. **B)** Evaluation of GLS isoform GAC expression and **C)** KGA expression in BT-20 cells upon FAHD1 KD. Mean of n = 8 biological replicates with SEM, also see Figure 5E. **D)** Levels of mitochondrial phosphoenolpyruvate-carboxykinase (mPEPCK) were measured *via* western blot in SCR and FAHD1 KD BT-20 cells which were culture for 48 h in limiting conditions. **E)** Protein expression of succinate-dehydrogenase (Complex II, SDH) and NADH:ubiquinone-oxidoreductase (mitochondrial complex I, *NDUFA9*) were analyzed in SCR and FAHD1 KD BT-20 cells. Significance threshold for students t-test: * $p \le 0.05$; ** $p \le 0.01$; *** $p \le 0.001$
